# Leukocyte glucose index as a novel biomarker for COVID-19 severity

**DOI:** 10.1038/s41598-022-18786-5

**Published:** 2022-09-02

**Authors:** Wendy Marilú Ramos-Hernández, Luis F. Soto, Marcos Del Rosario-Trinidad, Carlos Noe Farfan-Morales, Luis Adrián De Jesús-González, Gustavo Martínez-Mier, Juan Fidel Osuna-Ramos, Fernando Bastida-González, Víctor Bernal-Dolores, Rosa María del Ángel, José Manuel Reyes-Ruiz

**Affiliations:** 1 Unidad Médica de Alta Especialidad, Hospital de Especialidades No. 14, Centro Médico Nacional “Adolfo Ruiz Cortines”, Instituto Mexicano del Seguro Social (IMSS), 91897 Veracruz, México; 2 Escuela Profesional de Genética y Biotecnología, Facultad de Ciencias Biológicas, Universidad Nacional Mayor de San Marcos, Lima, 15081 Perú; 3 Department of Infectomics and Molecular Pathogenesis, Center for Research and Advanced Studies (CINVESTAV-IPN), 07360 Mexico City, Mexico; 4 Escuela de Medicina, Universidad Autónoma de Durango Campus Culiacán, 80050 Culiacán Rosales, México; 5 Laboratorio de Biología Molecular, Laboratorio Estatal de Salud Pública del Estado de México, 50130 Mexico City, State of Mexico Mexico; 6 Facultad de Medicina, Región Veracruz, Universidad Veracruzana, 91700 Veracruz, Mexico

**Keywords:** Virology, Microbiology, Predictive markers

## Abstract

The severity of coronavirus disease 2019 (COVID-19) quickly progresses with unfavorable outcomes due to the host immune response and metabolism alteration. Hence, we hypothesized that leukocyte glucose index (LGI) is a biomarker for severe COVID-19. This study involved 109 patients and the usefulness of LGI was evaluated and compared with other risk factors to predict COVID 19 severity. LGI was identified as an independent risk factor (odds ratio [OR] = 1.727, 95% confidence interval [CI]: 1.026–3.048, *P* = 0.041), with an area under the curve (AUC) of 0.749 (95% CI: 0.642–0.857, *P* < 0.0001). Interestingly, LGI was a potential risk factor (OR = 2.694, 95% CI: 1.575–5.283, *P*_corrected_ < 0.05) for severe COVID-19 in female but not in male patients. In addition, LGI proved to be a strong predictor of the severity in patients with diabetes (AUC = 0.915 (95% CI: 0.830–1), sensitivity = 0.833, and specificity = 0.931). The AUC of LGI, together with the respiratory rate (LGI + RR), showed a considerable improvement (AUC = 0.894, 95% CI: 0.835–0.954) compared to the other biochemical and respiratory parameters analyzed. Together, these findings indicate that LGI could potentially be used as a biomarker of severity in COVID-19 patients.

## Introduction

Since December 2019, coronavirus disease 2019 (COVID-19), caused by the Severe Acute Respiratory Syndrome Coronavirus 2 (SARS-CoV-2), has been rapidly spreading all over the world^1^. As of 5 July 2020, the COVID-19 pandemic caused more than 582 000 deaths and infected more than 11 million people in over 200 countries^2^.

The clinical spectrum of COVID-19 includes mild disease, severe pneumonia, multiple-organ failure, leading in some cases to death. Some risk factors associated with severe outcomes of SARS-CoV-2 infection are male gender, older age, and the presence of comorbidities such as cardiovascular disease, diabetes, hypertension, and obesity^3^. In turn, several studies have identified biomarkers for diabetes and other comorbidities^4^. These clinical features contribute to severe respiratory failure in COVID-19 patients, requiring their admission to the intensive care unit for mechanical ventilation^5^. During the inflammatory response in COVID-19 pneumonia, there is dysregulation of circulating biomarkers such as C-reactive protein (CRP), D-dimer, neutrophil-to-lymphocyte ratio (NLR), platelet-to-lymphocyte ratio (PLT), and blood urea nitrogen/creatinine ratio (BUN/Cr)^6–8^. These biochemical parameters, together with respiratory parameters: respiratory rate (RR), peripheral oxygen saturation (SpO_2_), partial pressure of oxygen (PaO_2_), the fraction of inspired oxygen (FiO_2_), and respiratory rate oxygenation (ROX) index, have been reported as indicators of the COVID-19 prognosis^9,10^.

Blood leukocytes and glucose are inexpensive, standard, and broadly used markers of inflammation^11^, and they are involved in the severe COVID-19^12,13^, where the inflammation inflicts multi-organ damage leading to organ failure^14^. In this regard, these parameters and the leukocyte glucose index (LGI), calculated from measurements of blood leukocytes count and glucose levels, have predictive values for acute myocardial infarction, coronary artery bypass grafting, and pneumonia after acute ischemic stroke^15–18^. Taken together, we hypothesized that LGI should be contemplated as another clinical tool for determining the severity of SARS-CoV-2 infection.

Therefore, in this study, we analyzed the clinical data of 109 patients to evaluate the potential of LGI as a novel predictor of COVID-19 severity and compared its usefulness with previously reported biochemical and respiratory markers.

## Results

### Patient characteristics

A total of 109 patients with confirmed COVID-19 were enrolled in the present study, where were including 36 severe and 73 non-severe COVID-19 cases. Two patients were previously excluded from the analysis due to their elevated blood glucose levels. The mean (SD) age and body mass index (BMI) in patients diagnosed with severe infection were 56.5 (15.2) years and 26.7 (3.1) kg/m^2^, respectively. Table 1 shows the baseline characteristics of all patients. There were no significant differences regarding age, weight, and BMI between severe and non-severe COVID-19 groups (*P*_*corrected*_ = 1).

**Table 1:** Clinical characteristics and laboratory findings in patients with non-severe and severe COVID-19.

Variable	Severe group (n = 36)	Non-severe group (n = 73)	*P* value
**Demographic characteristics**			
Gender, female	22 (61.11%)	28 (38.36%)	**0.041**
Age, years old	51.94 (16.3)	58.78 (14.26)	0.068
Height, meters	1.6 (0.07)	1.64 (0.08)	**0.007**
Weight, Kg	68 (9.59)	72.07 (8.83)	**0.020**
BMI, Kg/m^2^	26.62 (3.73)	26.77 (2.73)	0.548
**Comorbidities, n (%)**			
CKD	11 (30.56%)	15 (20.55%)	0.360
Diabetes	18 (50%)	29 (39.73%)	0.416
COPD	2 (5.56%)	7 (9.59%)	0.726
**Clinical and laboratory data**			
Onset of Symptom to Hospital admission, days	7.56 (3.59)	7.11 (5)	0.074
RR, beats per minute	32.92 (9.61)	23.29 (4.49)	< 0.0001*
SpO_2_, %	73.19 (21.33)	91.48 (9.02)	< 0.0001*
FiO_2_, %	45.22 (21.18)	31.3 (12.86)	** < 0.001**
SaO_2_, %	71.5 (19.38)	88.05 (8.72)	** < 0.0001**
PaO_2_, mmHg	66 (31.12)	91.03 (60.82)	**0.001**
HCO_3_^−^, mmol/L	17.61 (7.16)	25.22 (7.16)	< 0.0001*
pH	7.28 (0.14)	7.4 (0.08)	< 0.0001*
Diarrhea, n (%)	12 (33.33%)	2 (2.74%)	** < 0.0001**
Arthralgia, n (%)	11 (30.56%)	2 (2.74%)	< 0.0001*
Leukocytes, × 10^9^/L	14.1 (4.15)	10.09 (3.43)	< 0.0001*
Lymphocytes, × 10^9^/L	7.67 (5.72)	9.45 (7.98)	0.459
Neutrophils, × 10^9^/L	84.69 (7.13)	78.58 (11.94)	**0.018**
RBC, × 10^12^/L	3.54 (0.59)	3.48 (1.13)	0.430
Hemoglobin, g/dL	10.53 (2.55)	12.37 (2.34)	** < 0.001**
Glucose, mg/dL	204.67 (100.48)	156.15 (93.5)	**0.008**
NLR	15.25 (7.52)	10.36 (8.02)	0.001*
LGI	2.94 (1.63)	1.61 (1.17)	< 0.0001*
ROX index	6.74 (4.65)	15.12 (6.31)	< 0.0001*
PAFI, mmHg	174.77 (120.55)	317 (180.13)	< 0.0001*
SAFI, mmHg	191.95 (100.59)	336.5 (119.33)	< 0.0001*
CO-RADS 3	1 (2.8%)	15 (20.5%)	< 0.0001*
CO-RADS 4	5 (13.9%)	31 (42.5%)	
CO-RADS 5	30 (83.3%)	27 (37.0%)	

Data are presented as mean ± SD or number (%). Statistically significant *P* values (< 0.05) are highlighted in bold. Variables that remained significant after considering gender are indicated with asterisk in the *P* value (*).

*RR* respiratory rate, *BMI* body mass index, *COPD* chronic obstructive pulmonary disease, *CKD* chronic kidney disease, FiO_2_ fraction of inspired oxygen, SpO_2_ peripherical oxygen saturation, SaO_2_ oxygen saturation, PaO_2_ partial pressure of oxygen, HCO_3_^−^ arterial bicarbonate, *pH* potential hydrogen, *RBC* red blood cells, *NLR* neutrophil to lymphocyte ratio, *LGI* leukocyte glucose index, *ROX* index respiratory rate oxygenation index, *PAFI* PaO_2_/FiO_2_, *SAFI* SaO_2_/FiO_2_, *CO-RADS* the COVID-19 Reporting and Data System.

Gender was statistically associated with COVID-19 severity (*P* = 0.04); however, it did not remain significant when multiple hypothesis correction was performed (*P*_*corrected*_ = 1). A similar effect was observed in hematocrit (*P* = 0.001; *P*_*corrected*_ = 0.05), temperature (*P* = 0.003; *P*_*corrected*_ = 0.1), sodium (*P* = 0.006; *P*_*corrected*_ = 0.3), myalgia (*P* = 0.01; *P*_*corrected*_ = 0.5), and HR (*P* = 0.03; *P*_*corrected*_ = 1) (Supplementary Table S1online). The mean of days from onset of symptoms to hospital admission was seven days in both groups (*P*_*corrected*_ = 1). Regarding comorbidities, hypertension was the most frequent (57.8%), followed by diabetes (43.2%) in all the patients. There were no significant differences between the severe and non-severe groups regarding diabetes, CKD, hypertension, and COPD. The CO-RADS, a scoring system previously reported to be a predictor of COVID-19 severity, was showed a significant difference between both groups (*P*_*corrected*_ < 0.001). The leukocytes count, LGI, and FiO_2_ were significantly higher in the severe group while the ROX index, RR, SpO_2_, SAFI, PAFI, HCO_3_^-^, pH, SaO_2_, and hemoglobin (*P*_*corrected*_ < 0.05) were significantly lower. Diarrhea and arthralgia were symptoms significantly associated with severity being present in roughly 30% of severe patients. Levels of glucose, NLR, PaO_2_, neutrophils, RBC, and lymphocytes were found no significant between both groups, like other variables included in this study (Supplementary Table S1 online).

**Figure 1:**
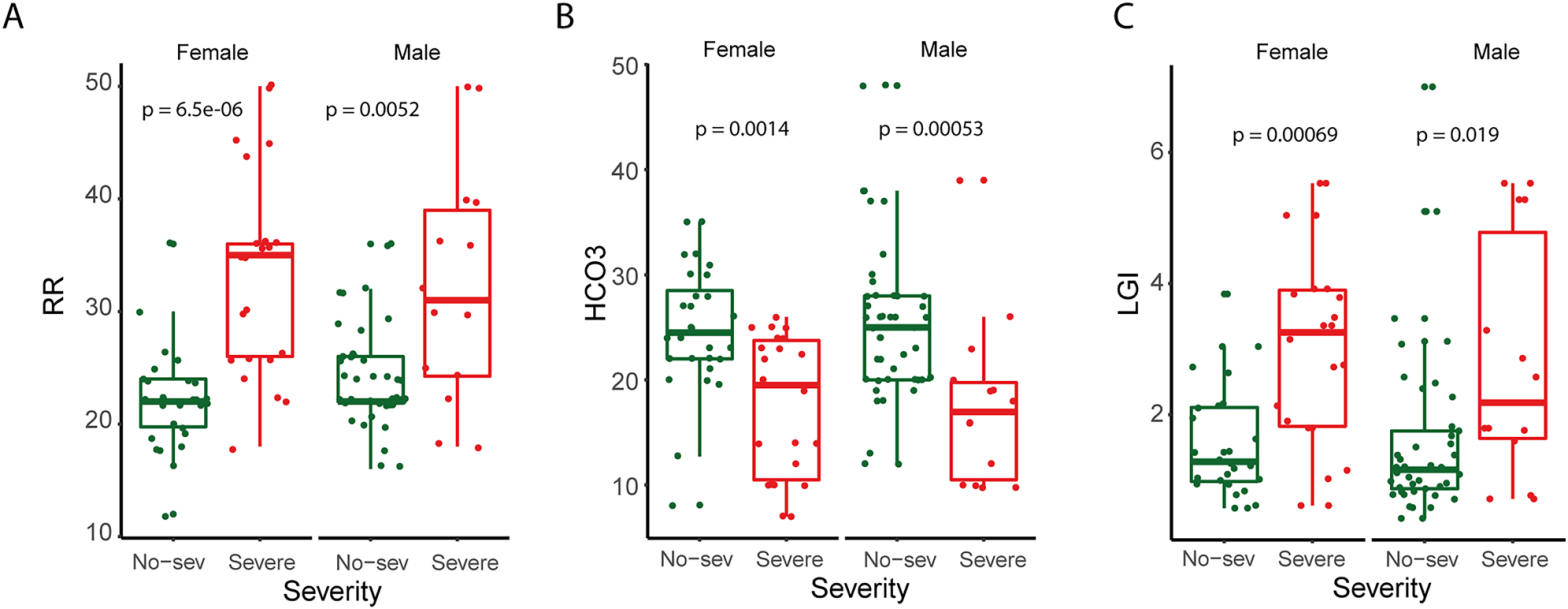
Clinical parameters associated with COVID-19 severity after adjusting gender. Box plot showing the difference of (**A**) RR, (**B**) HCO_3_^-^, and (**C**) LGI between non-severe and severe groups in female (left) and male (right) patients. Groups were compared using Mann U Whitney test, *P* < 0.05. RR: respiratory rate; HCO_3_^−^: arterial bicarbonate; LGI: leuko-glycemic index.

Since a significantly higher proportion of females with severe COVID-19 (61.11% vs. 38.36%, *P* = 0.04) was observed, we evaluated whether all 15 previous significant variables remained significant when data were adjusted by gender. In this regard, 12 variables remained significant in both male and female groups. Some of these variables, such as SAFI and PAFI, had been previously reported as predictors of severe COVID-19^19^. However, new variables such as RR, HCO3-, and LGI among others also were associated with the COVID-19 severity regardless the gender (Fig. 1A-C). Therefore, these results indicated that LGI was significantly higher in the severe group, independent of gender.

### Identification of candidate predictors for severe COVID-19

According to the previous outcomes, the 15 statistically significant characteristics were selected to perform univariate logistic regression analysis in patients with COVID-19. As shown in Table 2, the univariate analysis revealed that all of them were significant with a *P* < 0.001 and *P*_*corrected*_ < 0.05. We found that the variables with the highest OR [95% CI] values were diarrhea (17.749, 4.440–119.459, *P*_*corrected*_ < 0.005), arthralgia (15.619, 3.862–105.574, *P*_*corrected*_ = 0.009), and CO-RADS (5.494, 2.513–14.189, *P*_*corrected*_ = 0.001). LGI showed an OR of 1.923 [1.421–2.719] which was higher than the ORs [95% CI] from ROX index (0.765 [0.677–0.843]), SAFI (0.988 [0.983–0.993]), RR (1.220 [1.132–1.335]), PAFI (0.991 [0.986–0.995]), leukocytes (1.323 [1.171–1.531]), and hemoglobin (0.734 [0.608–0.871]) among others (Table 2).

**Table 2:** Risk factors associated with severity for COVID-19 patients.

Predictors	OR [95% CI]	*P* value	Adjusted *P* value
Diarrhea	17.75 [4.440–119.459]	< 0.001	**0.004**
Arthralgia	15.62 [3.862–105.574]	< 0.001	**0.009**
CO-RADS	5.494 [2.513–14.189]	< 0.0001	**0.001**
LGI	1.923 [1.421–2.719]	< 0.0001	**0.001**
Leukocytes, × 10^9^/L	1.323 [1.171–1.531]	< 0.0001	**0.0004**
RR, beats per minute	1.220 [1.132–1.335]	< 0.0001	** < 0.0001**
FiO_2_, %	1.051 [1.024–1.084]	< 0.001	**0.006**
PAFI, mmHg	0.991 [0.986–0.995]	0.0001	**0.001**
SAFI, mmHg	0.988 [0.983–0.993]	< 0.0001	** < 0.0001**
S_P_O_2_, %	0.9163 [0.876–0.950]	< 0.0001	** < 0.001**
SaO_2_, %	0.9161 [0.877–0.949]	< 0.0001	**0.0001**
HCO_3_^−^, mmol/L	0.847 [0.780–0.908]	< 0.0001	** < 0.001**
ROX index	0.765 [0.677–0.843]	< 0.0001	** < 0.0001**
Hemoglobin, g/dL	0.734 [0.608–0.871]	< 0.001	**0.010**
pH	0.0000258 [0.000000872–0.002]	< 0.0001	** < 0.001**

Univariable logistic regression analysis was performed using the 15 variables that had statistically significant difference when multiple hypothesis correction was performed (*P*_corrected_ < 0.05). Odds ratios (OR) and 95% Confidence Interval (CI 95%) are reported. Statistically significant *P* values (< 0.05) are highlighted in bold. *CO-RADS* the COVID-19 Reporting and Data System, *LGI* leukocyte glucose index, *RR* respiratory rate, FiO_2_ fraction of inspired oxygen, *PAFI* PaO_2_/FiO_2_, *SAFI* SaO_2_/FiO_2_, SaO_2_ oxygen saturation, HCO_3_^−^ arterial bicarbonate, *ROX* index respiratory rate oxygenation index.

In order to evaluate the predictive value of the parameters involved in the COVID-19 severity, ROC curves analysis was performed. The AUC and the optimal cut-off values of each variable are shown in Table 3. ROX index showed the highest AUC of 0.863 (95% CI 0.791–0.935, *P* < 0.000005) with an optimal cut-off value of 9.09 (sensitivity, 0.833; specificity, 0.780) while SAFI had an AUC of 0.816 (95% CI 0.7280.904, *P* < 0.000005) and their optimal cut-off value was 235 mmHg (sensitivity, 0.805; specificity, 0.712). RR (AUC = 0.809 [95% CI 0.712–0.907]) and SpO_2_ (AUC = 0.804 [95% CI 0.709–0.900]) also showed high values of AUC. PAFI, leukocytes, HCO_3_^-^, pH, and SaO_2_ showed an AUC higher than 0.750. Moreover, LGI had an AUC of 0.749 (95% CI 0.642–0.857, *P* = 0.00006) and an optimal cut-off value of 1.764 (sensitivity, 0.777; specificity, 0.726).

**Table 3:** Cut-off values of the risk factors for COVID-19 severity.

Parameters	AUC	95% CI	*P* value	Optimal cut-off	Sensitivity	Specificity
ROX index	0,8632	79.129 to 93.510	** < 0.0001**	9.0909	0.8333	0.7808
SAFI, mmHg	0.8165	72.823 to 90.494	** < 0.0001**	235	0.8055	0.7123
RR, beats per minute	0.8099	71.241 to 90.745	** < 0.0001**	30	0.6388	0.9041
S_P_O_2_, %	0.8049	70.976 to 90.020	** < 0.0001**	89	0.7777	0.7671
PAFI, mmHg	0.7949	70.271 to 88.709	**0.0001**	202.5	0.8055	0.7260
Leukocytes, × 10^9^/L	0.7931	69.914 to 88.723	** < 0.0001**	12	0.8055	0.7260
HCO_3_^-^, mmol/L	0.7838	69.136 to 87.636	** < 0.0001**	19	0.5833	0.8767
pH	0.7821	68.351 to 88.078	** < 0.0001**	7.33	0.7222	0.8219
SaO_2_, %	0.7684	65.779 to 87.911	** < 0.0001**	80	0.6944	0.7945
LGI	0.7498	64.202 to 85.759	** < 0.0001**	1.764	0.7777	0.7260
Hemoglobin, g/dL	0.7258	62.214 to 82.952	** < 0.001**	11.5	0.6944	0.7671
FiO_2_	0.7045	60.545 to 80.360	** < 0.001**	40	0.7777	0.5890

Statistically significant *P* values (< 0.05) are highlighted in bold. *RR* respiratory rate, SpO_2_ peripherical oxygen saturation, HCO_3_^−^ arterial bicarbonate, *pH* potential hydrogen, SaO_2_ oxygen saturation, *LGI* leukocyte glucose index, FiO_2_ fraction of inspired oxygen.

Interestingly, we identified that these predictors of severity differed in female and male patients infected with SARS-CoV-2. In the male group, the leukocytes (OR 1.359, 95% CI 1.145–1.696, *P*_*corrected*_ = 0.025), ROX index (OR 0.816, 95% CI 0.700–0.919, *P*_*corrected*_ < 0.05), and hemoglobin (OR 0.610, 95% CI 0.439–0.796, *P*_*corrected*_ = 0.013) showing an AUC higher than 0.750. On the contrary, the best predictors of severe COVID-19 in the female group with an AUC higher than 0.750 were ROX index (OR 0.704, 95% CI 0.553–0.828, *P*_*corrected*_ = 0.006), SaO_2_ (OR 0.869, 95% CI 0.783–0.932, *P*_*corrected*_ < 0.05), RR (OR 1.297, 95% CI 1.143–1.561, *P*_*corrected*_ = 0.011), HCO_3_^−^ (OR 0.838, 95% CI 0.735–0.926, *P*_*corrected*_ < 0.05), and LGI (OR 2.694, 95% CI 1.575–5.283, *P*_*corrected*_ < 0.05) (Supplementary Table S2online). This analysis showed that LGI could better distinguish severity in female than male patients with SARS-CoV-2 infection.

**Figure 2:**
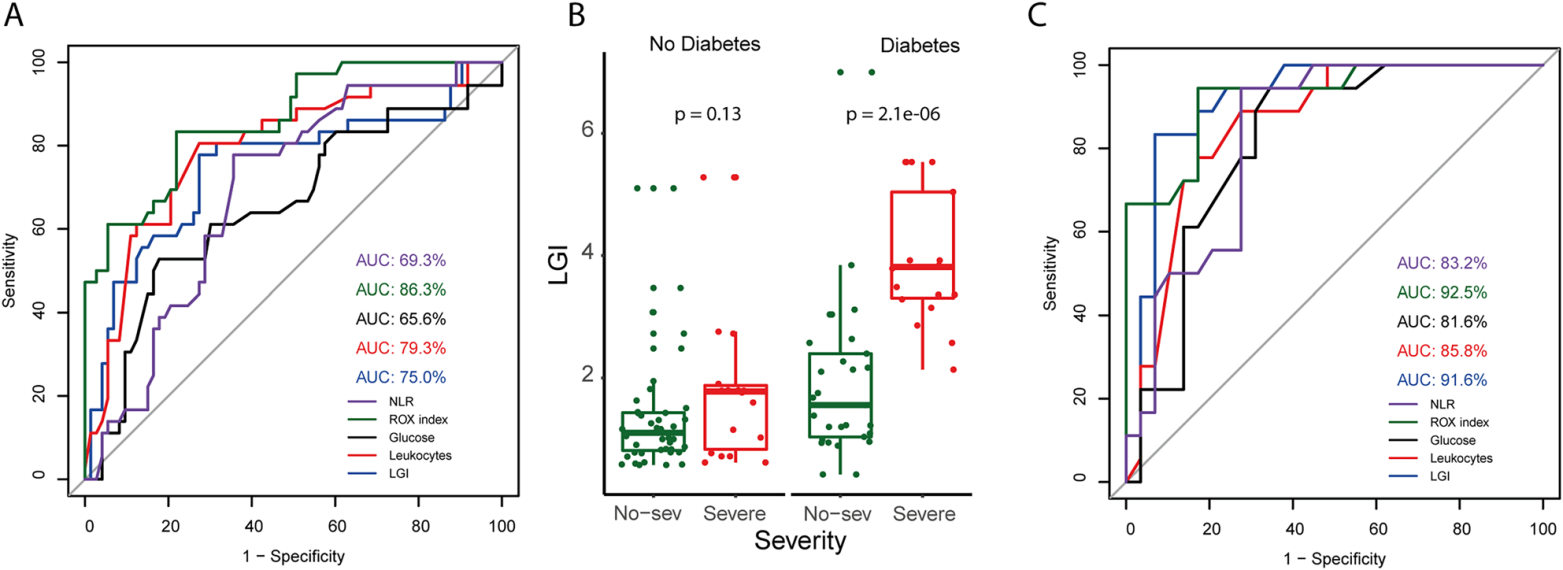
ROC analysis and LGI levels in diabetic patients with severe COVID-19. Comparison of ROC curve of NLR, ROX index, glucose, leukocytes, and LGI for predicting severe COVID-19 in all patients (**A**) or patients with diabetes (**C**). LGI levels in patients with non-severe or severe COVID-19 after adjusting the data for diabetes (**B**). The AUC of NLR, ROX index, glucose, leukocytes, and LGI were represented with purple, green, black, red, and blue lines, respectively. AUC: Area under the curve; NLR: neutrophil to lymphocyte ratio; LGI: leuko-glycemic index.

Since LGI was statistically significant between non-severe and severe groups, their predictor value was compared with other blood parameters (NLR, glucose, ROX index, leukocytes), previously reported as predictors of disease severity^6,10,20–22^. The AUC of LGI was higher than that for glucose and leukocytes, in contrast with the ROX index and NLR (Fig. 2A). Although, diabetes was not related to COVID-19 severity (*P* = 0.41) (Table 1), LGI was statistically higher in the severe group of patients with diabetes (*P* < 0.0001) while no significance was found in patients without diabetes (*P* = 0.13) (Fig. 2B). To further explore the usefulness of LGI, a ROC analysis for LGI and other blood parameters was performed in patients with diabetes. The results obtained demonstrated that LGI had an AUC higher than when all patients were considered. The AUC of LGI was 0.915 (95% CI 0.830–1, [sensitivity, 0.833; specificity, 0.931]) being higher than for the AUC of NLR (0.832), glucose (0.816), and leukocytes (0.858) and almost equal to the ROX index (0.925) (Fig. 2C). Although the optimal cut-off of LGI was different between the patients with diabetes alone (3.15) and all patients (1.764), the cut-off points for ROX index, RR, leukocytes, pH, and FiO_2_ were the same in both groups. (Supplementary Table S3 online). This finding suggested that LGI is a strong predictor of COVID-19 severity in diabetes patients.

### LGI as a strong predictor of the COVID-19 severity

A multivariate logistic regression with stepwise procedure was performed to determine the predictors of COVID-19 severity, considering all 15 variables with statistically significant difference (*P* < 0.05), obtained from the univariate analysis. Interestingly, the LGI remained significant together with other 4 variables (Table 4). Results showed that LGI was independent risk factor for severe COVID-19 with a OR value of 1.727 (95% CI 1.026–3.048; *P* = 0.041). Other risk factors for severe infection included RR (OR = 1.165; 95% CI 1.014–1.376; *P* = 0.044), FiO_2_ (OR = 1.070; 95% CI 1.010–1.150; *P* = 0.034), HCO_3_^−^ (OR = 0.862; 95% CI 0.741–0.969; *P* = 0.026), and hemoglobin (OR = 0.505; 95% CI 0.315–0.713; *P* = 0.0007) (Table 4).

**Table 4:** Independent predictors for COVID-19 severity.

Predictor	OR [95% CI]	*P* value
Hemoglobin	0.505 [0.315—0.713]	** < 0.001**
HCO_3_^-^	0.862 [0,741—0.969]	**0.026**
FiO_2_	1.070 [1.010—1.150]	**0.034**
LGI	1.727 [1.026—3.048]	**0.041**
RR	1.165 [1.014—1.376]	**0.044**
SaO_2_	0.940 [0.861—1.012]	0.125
CO-RADS	2.825 [0.828—12.736]	0.126
SpO_2_	0.957 [0.898—1.010]	0.133

Candidate predictors with statistically significant difference (*P* < 0.05) in univariate logistic analysis were included a multivariable logistic regression. Odds ratios (OR) and 95% Confidence Interval (CI 95%) are reported. Statistically significant *P* values (< 0.05) are highlighted in bold. HCO_3_^−^: arterial bicarbonate; FiO_2_: fraction of inspired oxygen; LGI: leukocyte glucose index; RR: respiratory rate; SaO_2_: oxygen saturation; CO-RADS: the COVID-19 Reporting and Data System; SpO_2_: peripherical oxygen saturation.

### The addition of RR and SaO2 to LGI improved the predictive value for severe COVID-19

**Figure 3:**
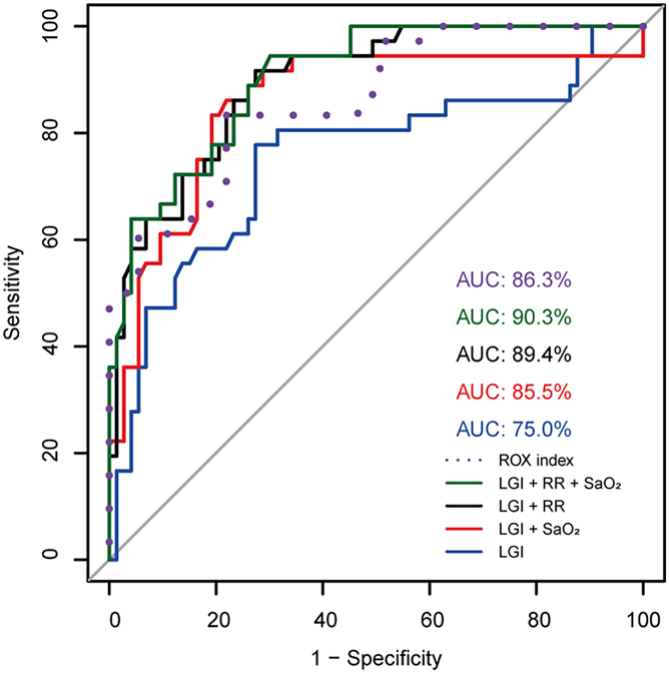
ROC analysis of LGI associated with other biomarkers AUC comparison of ROX index (dashed line), LGI (blue line), RR in combination with LGI (black line), SaO_2_ in combination with LGI (red line), and the combination of SaO_2_, RR, and LGI (green line). AUC: Area under the curve; LGI: leuko-glycemic index; RR: respiratory rate; SaO_2_: oxygen saturation.

In other to determine the utility of RR and SaO_2_ in addition to LGI for the prediction of COVID-19 patients at high risk of progression to severe disease, a ROC curve analysis was performed (Fig. 3). The model including RR and LGI improved the predictive performance for COVID-19 severity showing an AUC of 0.894 [95% CI 0.835–0.958]. This AUC was higher compared to the ROX index (AUC = 0.863, 95% CI 0.791–0.935). The inclusion of SaO_2_ with LGI showed an AUC of 0.855 [95% CI 0.769–0.941]; however, it was lower compared to the AUC of the ROX index. Finally, the AUC of RR and SaO_2_, in addition to LGI, resulted in a considerable improvement of the predictive performance (AUC = 0.903, 95% CI 0.847–0.958) compared to LGI alone (AUC = 0.749) and ROX index (Fig. 3).

## Discussion

The current study proposes a novel factor to predict severity of patients with COVID-19. High levels of leukocytes and glucose have been associated with inflammatory response, acute myocardial infarction, and pneumonia in acute ischemic stroke^16–18^. The SARS-CoV-2 infection produces an aggressive inflammatory response strongly implicated in pneumonia and multi-organ damage leading to organ failure, especially in the cardiac and nervous systems^14,23^. The LGI, which considers leukocytes count and blood glucose level, has been shown to have a predictive value in coronary artery disease, a pathology associated with an increased risk of several clinical complications in COVID-19 patients^15,24^. Hence, this study explored the usefulness of LGI in the prediction of severe COVID-19.

We found a significantly higher proportion of females with severe COVID-19. After the multiple hypothesis correction, the gender was not significantly different between the severe and non-severe groups. This observation could be due to the relatively small sample size. A multiple hypothesis testing correction is recommended to avoid false positives when multiple variables are analyzed. For example, a previous study used the Benjamini–Hochberg procedure as they analyzed several biomarkers^4^. We applied the Bonferroni correction, a more stringent procedure, to discard the slightly significant variables. Moreover, a clear explanation that men have a higher risk of severe COVID-19 than women has not been established yet^25^. Biological and immunological pathways, socio-behavioral, and cultural aspects could be involved in COVID-19 severity in a sex-dependent manner^26^.

The results showed that the most common symptoms found were the same as reported previously^27^, including arthralgia and diarrhea. Comorbidities such as diabetes were not associated with the COVID-19 severity, agreeing with a previous multi-center study^28^. Although symptoms such as diarrhea and arthralgia, and CO-RADS were three main predictors for admission (univariate analysis), they could not prove to be effective severity indicators. The ROX index showed that highest AUC (0.863); however, leukocytes, LGI, RR, FiO2, FAPI, SAFI, SaO2, HCO3, ROX index, and hemoglobin were also candidates to predict COVID-19 severity. A retrospective study reported the potential predictive value of ROX index for deterioration in 186 patients with COVID-19 (AUC = 0.848)^10^. Even though the ROX index is a simple model based solely on three respiratory parameters ([SpO2/FiO2]/RR)^10^ and LGI can be easily calculated with two biochemical parameters, our results suggests that both parameters are good predictors. The ROX index had greater predictive validity than LGI (AUC = 0.749); however, the multivariate analysis revealed that the ROX index was not an independent factor that aided in predicting the disease severity. This could probably be because the other variables considered in the multivariate analysis were sufficient to predict the COVID-19 severity and the ROX index was not adding significative information. This suggests that ROX index is a good sole predictor, but a pair of biomarkers could have a better performance as shown in the case of LGI + RR (Fig. 3). In contrast, RR, FiO2, hemoglobin, HCO3-, and LGI remained after the stepwise procedure indicating that they are independent predictors of severe disease conditions. RR and FiO_2_ (involved in calculating ROX index), hemoglobin, and HCO_3_^-^ have been reported to be associated with COVID-19 severity^10,29,30^. Nevertheless, the OR for the LGI was higher than the other parameters when were considered all (1.727, 95% IC 1.026–3.048) or only female patients (2.694, 95% IC 1.575–5.283). As an independent risk factor, LGI had the highest AUC of 0.915 (95% IC 0.830–1), with a sensitivity of 0.833 and a specificity of 0.931, in patients with diabetes. This result may be due to the blood glucose levels correlate with clinical outcomes in patients with COVID-19 and pre-existing diabetes^13,20^. Furthermore, high glucose levels in monocytes directly promote cytokine production, viral replication, subsequent T cell dysfunction, and lung epithelial cell death^31^. Leukocytes > 5.37 × 10^9^/L count has been associated with a higher risk of death and shorter survival time in patients with COVID-19 and diabetes^12^. Thus, the link between blood glucose levels and leukocytes counts in the COVID-19 severity suggests that LGI should be analyzed as a new predictor. Interestingly, the LGI showed to be an excellent predictor for COVID-19 severity in patients with diabetes, being higher than the AUC of D-dimer (0.909) for mortality risk^32^. The optimal LGI cut-off for severe COVID-19 was 1.764 in all patients, while in patients with this condition and diabetes was 3.15. This cutoff was similar to a previous study that reported an optimal cut-off of 2 for LGI in postoperative coronary artery bypass grafting^15^; indicating that high levels of LGI are associated with different diseases. Due to the predominant respiratory features of COVID-19 and that RR is a physiological variable associated with a significant increase in the odds ratio for the ROX index^10^, we analyzed the combined effect of RR and LGI (LGI + RR) in predicting for severity. The AUC of LGI + RR (0.894) was higher compared with the combined effect observed in other studies such as the combination of (a) procalcitonin (PCT), CRP and NLR (AUC = 0.84)^33^; (b) NLR, platelet count and CRP (AUC = 0.774)^6^; and (c) CD4^+^ T cell count, NLR, and D-dimer (AUC = 0.865)^34^. Therefore, LGI + RR showed to be a strong predictor and its easy implementation makes it a valuable marker.

Until now, there has been a paucity of research that has combined respiratory and biochemical parameters to find a strong predictor of COVID-19, particularly in the severity The models from Zhou, King, Ji, Haimovich, and Altschul, with AUC of 0.862, 0.79, 0.91, 0.89, and 0.798, respectively, have also been suggested to predict the COVID-19 outcome^35–39^. Nevertheless, their candidate predictors require a complicated composite of patient-related risk factors, which makes challenging to operationalize. Moreover, machine learning-based predictors are not straightforward to be widely implemented^40,41^. The predictive model from Patel et al. was performed considering only 5 of 72 variables for predicting intensive care unit (ICU) need (CRP, D-dimer, PCT, SpO_2_, and RR), obtaining an AUC of 0.79^40^. In contrast, the Cheng et al. model identified 20 predictive variables to ICU transfer in hospitalized COVID-19 patients^41^. These variables were related to progressive respiratory failure (RR and SaO_2_), markers of systemic inflammation (leukocytes count and CRP), among others, resulting in an AUC of 0.799^41^. RR was a significant variable^40^ and had the highest predictive value, followed by leukocytes^41^. Hence, these findings confirm the usefulness of leukocytes and RR in predicting COVID-19 severity, supporting the result obtained here, where the predictive value of LGI increased when the effect of RR and LGI were combined, LGI + RR.

The severe COVID-19 is associated with an uncontrolled inflammatory response^42^. In this sense, the NLR, blood urea nitrogen/creatinine ratio, and platelet-lymphocyte ratio (PLR) are reliable indicators of the COVID-19 severity^6,8,43^. Nevertheless, we not find any positive association between these parameters and severe COVID-19. Regarding the NLR, a most recent study conducted in 76 patients with COVID-19 revealed that this biochemical parameter (NLR ≥ 3.59) was not an independent risk factor for death^33^. Xu et al. study may suggest why our finding did not show a positive correlation between NLR and severe disease. Lymphocyte, neutrophil, and platelet counts, used to calculate NLR and PLR, are part of the system immune-inflammation index (SII), a good index to reflect the immune response and systemic inflammation^44^. However, these parameters were also not associated with the COVID-19 severity in this study. NLR is an independent risk factor for severe disease or mortality, with an AUC of 0.737 (Shang et al.), 0.69 (Xu et al.), and 0.87 (Ok et al.)^6,8,33^. Thus, a simple index as LGI (two biochemical parameters: blood leukocytes and glucose) in combination with RR (respiratory parameter), LGI + RR (AUC = 0.894) can outperform inflammatory biomarkers in predicting the severe COVID-19. Glucose is the primary energy source, and its homeostasis is maintained by pancreatic β cells through insulin secretion. A recent study demonstrated that the β-cells were permissive to SARS-CoV-2 infection and replication, affecting glucose responsiveness^45^. Furthermore, high glucose highly induces viral replication and cytokine production during SARS-CoV-2 infection. Therefore, targeting glucose metabolism may offer a new practical antiviral approach^31,46^. Other molecules such as HDL-cholesterol and triglycerides, low and high concentrations, respectively, are strong predictors of a severe course of the COVID-19^47^. Since the host-inflammatory response and the regulation of metabolism are intimately connected with the COVID-19 severity, LGI (blood leukocytes count and glucose levels) appears to be a potent risk biomarker.

This study has limitations: (1) the study had a retrospective design and was conducted at a single center; (2) the sample size was small, which may affect the generalization of the results due to the limitation of enrolled patients. Although this study included a small sample, the results could be compared to other studies, with a similar number of patients, performed to determine clinical predictors for COVID-19 outcomes^9,33,34^. Nevertheless, the results of this study should be validated with additional studies or future efforts focused on the prospective analyses to strengthen our understanding of the predictive utility of LGI and LGI + RR. Other limitations are: (3) Since pharmacological therapies implemented during the hospitalization may affect the biochemical and respiratory parameters, the laboratory and clinical findings on the day of admission were selected to minimize the adverse impact of subsequent treatments; and (4) The respiratory and biochemical factors were not monitored regularly, thus, could not longitudinally evaluated the association between the dynamic changes of the factors and the severity during the disease course.

On the other hand, the current study had some strengths points. First, the evidence obtained on LGI as a biomarker for COVID-19 severity. Second, this study considered patients from Mexico, while other studies are based on cases from different countries, which is relevant since the outcomes for COVID-19 are known to be dependent on demographics^40^.

In conclusion, we demonstrate that values of just two or three features, namely, leukocytes, glucose, and RR, can predict the severe disease accurately obtained from all 51 variables included in this study. LGI and LGI + RR could serve as independent risk factors for predicting the COVID-19 severity and as an objective tool to support clinicians in their decision. These findings need to be further validated in a larger population of multi-center study.

## Methods

### Study design and participants

We conducted a retrospective observational study at a third level reference hospital (Unidad Médica de Alta Especialidad (UMAE), Hospital de Especialidades No. 14, Centro Médico Nacional (CMN) “Adolfo Ruiz Cortines”, Mexican Social Security Institute (IMSS)) from Veracruz, Mexico. A total of 109 COVID-19 patients with a positive nucleic acid test for SARS-CoV-2 from April to July 2020 were included. The clinical sample collection, processing, and COVID-19 testing for all patients were based on WHO guidelines^48^.

The COVID-19 patients who met any of the following criteria were excluded from the study: (1) pregnancy patients; (2) patients ages ≤ 18 years; (3) patients having severe medical conditions, including chronic renal dysfunction, malignant tumor, acquired immune deficiency syndrome, and liver cirrhosis; (4) patients with elevated blood glucose levels (> 600 mg/dL)^49^; and (5) patients with essential information deficits. Given the retrospective nature of the research, the Research Ethical Committee of the UMAE, Hospital de Especialidades No. 14, CMN “Adolfo Ruiz Cortines” from Mexican Social Security Institute (Registry code: CONBIOÉTICA-30-CEI-003–20,180,412; COFEPRIS 17CI30193067) approved the wavier for informed consent.

Disease severity was defined according to “Diagnosis and Treatment Protocol for Novel Coronavirus Pneumonia, issued by the Chinese Centers for Disease Control and Prevention^50^. Patients with a confirmed diagnosis of COVID-19 were classified into four types: (1) mild, patients with slight clinical symptoms and no imaging finding of pneumonia; (2) moderate, patients with fever and respiratory symptoms, and signs of pneumonia on radiologic assessment; (3) severe, patients met any of the following criteria (a. shortness of breath, RR ≥ 30 times/min; b. oxygen saturation ≤ 93% at rest; and c. partial pressure of oxygen (PaO_2_)/fraction of inspired oxygen (PaO_2_/FiO_2_ ≤ 300 mmHg); d. pulmonary imaging showing the significant progression of lesion > 50% within 24 to 48 h); and (4) critical, patients showing any of the following conditions (respiratory failure requires mechanical ventilation, shock, combined with other organ failure requires intensive care and treatment. For further analysis in this study, the patients were grouped as “non-severe” (classified as mild or moderate type) and “severe” (classified as severe or critical type) according to other reports where some factors were used to predict severe COVID-19^34,51^.

### Data collection

All data from the patients meeting the inclusion criteria were abstracted from the electronic medical records. Clinical parameters included age, sex, comorbidities, signs and symptoms, laboratory results, and vital signs were collected on admission.

### Definitions

All the biochemical parameters were determined at the hospital admission of the patients. The leukocyte glucose index (LGI) is defined as the product between blood leukocytes counts and glucose levels divided by 1000^15–18^. NLR was calculated by dividing the total absolute neutrophil counts over total lymphocyte counts^33^. PAFI was obtained as the ratio between partial pressure of oxygen (PaO_2_) and the fraction of inspired oxygen (FiO_2_)^19,52^. SAFI was determined as the ratio between the oxygen saturation (SaO_2_) and the FiO_2_^19,52^. The respiratory rate oxygenation (ROX) index was calculated using the following formula: ROX index = (SaO_2_/FiO_2_)/respiratory rate (RR)^53^. The COVID-19 Reporting and Data System (CO-RADS) is a categorical assessment scheme that includes a five-point scale for chest computed tomography in patients suspected of having COVID-19^54^.

### Statistical analysis

The data are presented as the mean ± standard deviations (SD) or numbers and frequencies (%). The Shapiro–Wilk test was used to evaluate the normality assumption of quantitative variables. We considered the standardized normal probability plots to analyze the dispersion and assess non-normality. The Chi-square test was performed to evaluate associations between categorical variables. Mann-Whiney U test and Kruskal–Wallis test were used to compare numerical variables between groups. Spearman’s rank correlation test was used to determine the correlations between continuous variables. Univariate and multivariate logistic regression analyses were performed to determine the variables affecting the severity of COVID-19. For multivariate logistic regression, a step-wise method was considered. The Odds Ratios (OR) were estimated with 95%CI. Receiver Operator Characteristic (ROC) curves were performed to evaluate and compare the Area Under the Curve (AUC) of LGI and other relevant variables associated with COVID-19 severity (adjusted *P* < 0.05). A predictor with an AUC above 0.7 was considered valuable, whereas an AUC between 0.8 and 0.9 indicated good diagnostic accuracy.

Logistic regression models were used to generate receiver operative curves for the variables of LGI, LGI + RR, LGI + SaO2, and LGI + RR + SaO2. We defined statistical significance as *P* < 0.05. *P* values were adjusted using the Bonferroni corrections (*P *_*corrected*_) to compensate the effect of multiple hypothesis testing. The variables were filtered using 0.05 as a significance cut-off^55^. The optimal cut-off points were determined considering the Youden index by showing the trade-off between sensitivity and specificity. All statistical analyses were performed with R v4.03 statistical software (R Foundation, Vienna, Austria).

## Ethics approval

This study was approved (code number R-2020-3001-068) by the Ethics Committee of the Unidad Médica de Alta Especialidad, Hospital de Especialidades No. 14, CMN “Adolfo Ruiz Cortines” Mexican Social Security Institute (IMSS). Including the exemption of the requirement for informed consent. The study was compliant with the Declaration of Helsinki. We certify that all protocols and methods are carried out under relevant guidelines and regulations. Due to Mexican laws, the research team cannot share the complete database used for the current paper. Since the number of patients included in this study was limited, the data could contain potentially identifying or sensitive patient information. Nevertheless, other researchers who meet the criteria may request access to the minimal data set underlying the results under request at the Ethics Committee.

## Supplementary Information

Supplementary Information.

## Data Availability

The datasets generated and analyzed during the study are available from the corresponding author upon reasonable request.

## References

[1] Wang, X., Zhou, Q., He, Y., et al. 2020. Nosocomial outbreak of 2019 Novel Coronavirus Pneumonia in Wuhan, China. European Respiratory Journal 2020; https://erj.ersjournals.com/content/early/2020/04/08/13993003.00544-2020. Accessed 31 May 2020.

[2] Erkhembayar R, Dickinson E, Badarch D (2020). Early policy actions and emergency response to the COVID-19 pandemic in Mongolia: Experiences and challenges. Lancet Glob. Health.

[3] Zhou F, Yu T, Du R (2020). Clinical course and risk factors for mortality of adult inpatients with COVID-19 in Wuhan, China: A retrospective cohort study. Lancet.

[4] Iyengar MF, Soto LF, Requena D (2020). Tear biomarkers and corneal sensitivity as an indicator of neuropathy in type 2 diabetes. Diabetes Res. Clin. Pract..

[5] Grasselli G, Zangrillo A, Zanella A (2020). Baseline characteristics and outcomes of 1591 patients infected with SARS-CoV-2 admitted to ICUs of the lombardy region, Italy. JAMA.

[6] Shang W, Dong J, Ren Y (2020). The value of clinical parameters in predicting the severity of COVID-19. J. Med. Virol..

[7] Bhargava A, Fukushima EA, Levine M (2020). Predictors for severe COVID-19 infection. Clin. Infect. Dis..

[8] Ok F, Erdogan O, Durmus E, Carkci S, Canik A (2021). Predictive values of blood urea nitrogen/creatinine ratio and other routine blood parameters on disease severity and survival of COVID-19 patients. J. Med. Virol..

[9] Gu Y, Wang D, Chen C (2021). PaO2/FiO2 and IL-6 are risk factors of mortality for intensive care COVID-19 patients. Sci. Rep..

[10] Prower E, Grant D, Bisquera A (2021). The ROX index has greater predictive validity than NEWS2 for deterioration in Covid-19. EClinicalMed..

[11] Lin H-Y, Zhang X-J, Liu Y-M, Geng L-Y, Guan L-Y, Li X-H (2021). Comparison of the triglyceride glucose index and blood leukocyte indices as predictors of metabolic syndrome in healthy Chinese population. Sci. Rep..

[12] Huang J, Zhu L, Bai X (2020). Multidimensional analysis of risk factors for the severity and mortality of patients with COVID-19 and diabetes. Infect Dis. Ther..

[13] Zhu L, She Z-G, Cheng X (2020). Association of blood glucose control and outcomes in patients with COVID-19 and pre-existing type 2 diabetes. Cell Metab..

[14] Tay MZ, Poh CM, Rénia L, MacAry PA, Ng LFP (2020). The trinity of COVID-19: immunity, inflammation and intervention. Nat. Rev. Immunol..

[15] Seoane, L. A., Espinoza, J. C., Burgos, L., *et al.* Prognostic value of the leukoglycaemic index in postoperative of coronary artery bypass grafting. *Eur. Heart J.* 2018; 39. 10.1093/eurheartj/ehy565.P1242. Accessed 16 Aug 2021.

[16] You S, Ou Z, Zhang W (2019). Combined utility of white blood cell count and blood glucose for predicting in-hospital outcomes in acute ischemic stroke. J. Neuroinflammation.

[17] Modan B, Schor S, Shani M (1975). Acute myocardial infarction: Prognostic value of white blood cell count and blood glucose level. JAMA.

[18] Ishihara M, Kojima S, Sakamoto T (2006). Usefulness of combined white blood cell count and plasma glucose for predicting in-hospital outcomes after acute myocardial infarction. Am. J. Cardiol..

[19] Guisado-Vasco, P., Valderas-Ortega, S., Carralón-González, M. M., *et al.* Clinical characteristics and outcomes among hospitalized adults with severe COVID-19 admitted to a tertiary medical center and receiving antiviral, antimalarials, glucocorticoids, or immunomodulation with tocilizumab or cyclosporine: A retrospective observational study (COQUIMA cohort). EClinicalMedicine 2020; 28. https://www.thelancet.com/journals/eclinm/article/PIIS2589-5370(20)30335-7/abstract. Accessed 30 August 2021. 10.1016/j.eclinm.2020.100591 PMC7557296 33078138

[20] Wu J, Huang J, Zhu G (2020). Elevation of blood glucose level predicts worse outcomes in hospitalized patients with COVID-19: A retrospective cohort study. BMJ Open Diabetes Res. Care.

[21] Velavan TP, Meyer CG (2020). Mild versus severe COVID-19: laboratory markers. Int. J. Infect. Dis..

[22] Mueller AA, Tamura T, Crowley CP (2020). Inflammatory biomarker trends predict respiratory decline in COVID-19 patients. Cell Rep. Med..

[23] Josephson SA, Kamel H (2020). Neurology and COVID-19. JAMA.

[24] Ganjali, S., Bianconi, V., Penson, P. E., *et al.* Commentary: Statins, COVID-19, and coronary artery disease: Killing two birds with one stone. Metabolism—Clinical and Experimental 2020; 113. https://www.metabolismjournal.com/article/S0026-0495(20)30239-0/fulltext. Accessed 9 Sep 2021. 10.1016/j.metabol.2020.154375 PMC7511211 32976855

[25] Jin J-M, Bai P, He W (2020). Gender differences in patients with COVID-19: Focus on severity and mortality. Front. Public Health.

[26] Salvati L, Biagioni B, Vivarelli E, Parronchi P (2020). A gendered magnifying glass on COVID-19. Clin Mol Allergy.

[27] Wang K, Qiu Z, Liu J (2020). Analysis of the clinical characteristics of 77 COVID-19 deaths. Sci Rep.

[28] Legris P, Vaillard L, Nonciaux C (2021). Diabetes is not associated with COVID-19-related mortality in older institutionalized people. Diabetes Metab.

[29] Rahman MA, Shanjana Y, Tushar MI (2021). Hematological abnormalities and comorbidities are associated with COVID-19 severity among hospitalized patients: Experience from Bangladesh. PLoS ONE.

[30] Tao Z, Xu J, Chen W (2021). Anemia is associated with severe illness in COVID-19: A retrospective cohort study. J. Med. Virol..

[31] Codo, A. C., Davanzo, G. G., Monteiro L de B., et al. Elevated glucose levels favor SARS-CoV-2 infection and monocyte response through a HIF-1α/Glycolysis-Dependent Axis. Cell Metabolism 32:437–446.e5 (2020). 10.1016/j.cmet.2020.07.007 PMC7367032 32697943

[32] He X, Yao F, Chen J (2021). The poor prognosis and influencing factors of high D-dimer levels for COVID-19 patients. Sci. Rep..

[33] Xu J, Xu C, Zhang R (2020). Associations of procalcitonin, C-reaction protein and neutrophil-to-lymphocyte ratio with mortality in hospitalized COVID-19 patients in China. Sci. Rep..

[34] Song C-Y, Xu J, He J-Q, Lu Y-Q (2020). Immune dysfunction following COVID-19, especially in severe patients. Sci. Rep..

[35] Haimovich AD, Ravindra NG, Stoytchev S (2020). Development and validation of the quick COVID-19 severity index: A prognostic tool for early clinical decompensation. Ann. Emerg. Med..

[36] Zhou Y, He Y, Yang H (2020). Development and validation a nomogram for predicting the risk of severe COVID-19: A multi-center study in Sichuan, China. PLOS One.

[37] Ji D, Zhang D, Xu J (2020). Prediction for progression risk in patients with COVID-19 pneumonia: The CALL score. Clin. Infect. Dis..

[38] Altschul DJ, Unda SR, Benton J (2020). A novel severity score to predict inpatient mortality in COVID-19 patients. Sci Rep.

[39] Knight SR, Ho A, Pius R (2020). Risk stratification of patients admitted to hospital with covid-19 using the ISARIC WHO clinical characterisation protocol: Development and validation of the 4C Mortality Score. BMJ.

[40] Patel D, Kher V, Desai B (2021). Machine learning based predictors for COVID-19 disease severity. Sci Rep.

[41] Cheng F-Y, Joshi H, Tandon P (2020). Using machine learning to predict ICU transfer in hospitalized COVID-19 patients. J. Clin. Med..

[42] Galván-Peña, S., Leon, J., Chowdhary, K., *et al.* Profound Treg perturbations correlate with COVID-19 severity. PNAS, 118. https://www.pnas.org/content/118/37/e2111315118. Accessed 11 September 2021. 10.1073/pnas.2111315118 PMC8449354 34433692

[43] Yang A-P, Liu J, Tao W, Li H (2020). The diagnostic and predictive role of NLR, d-NLR and PLR in COVID-19 patients. Int. Immunopharmacol..

[44] Huang H, Liu Q, Zhu L (2019). Prognostic value of preoperative systemic immune-inflammation index in patients with cervical cancer. Sci. Rep..

[45] Müller JA, Groß R, Conzelmann C (2021). SARS-CoV-2 infects and replicates in cells of the human endocrine and exocrine pancreas. Nat. Metab..

[46] Ardestani A, Azizi Z (2021). Targeting glucose metabolism for treatment of COVID-19. Sig. Transduct. Target Ther..

[47] Masana L, Correig E, Ibarretxe D (2021). Low HDL and high triglycerides predict COVID-19 severity. Sci. Rep..

[48] World Health Organization. Laboratory testing for coronavirus disease (COVID-19) in suspected human cases: interim guidance, 19 March 2020. World Health Organization, 2020. https://apps.who.int/iris/handle/10665/331501. Accessed 18 September 2021.

[49] Liang Y, Wanderer J, Nichols JH, Klonoff D, Rice MJ (2017). Blood gas analyzer accuracy of glucose measurements. Mayo Clin. Proc..

[50] Diagnosis and Treatment Protocol for COVID-19 (Trial Version 7). Available at: http://en.nhc.gov.cn/2020-03/29/c_78469.htm. Accessed 11 September 2021.

[51] Li Y, Shang K, Bian W (2020). Prediction of disease progression in patients with COVID-19 by artificial intelligence assisted lesion quantification. Sci. Rep..

[52] Swigris JJ, Zhou X, Wamboldt FS (2009). Exercise peripheral oxygen saturation (Spo2) accurately reflects arterial oxygen saturation (Sao2) and predicts mortality in systemic sclerosis. Thorax.

[53] Raoof S, Nava S, Carpati C, Hill NS (2020). High-flow, noninvasive ventilation and awake (Nonintubation) proning in patients with coronavirus disease 2019 with respiratory failure. Chest.

[54] Prokop M, van Everdingen W, van Rees VT (2020). CO-RADS: a categorical CT assessment scheme for patients suspected of having COVID-19—definition and evaluation. Radiology.

[55] Benjamini Y, Hochberg Y (1995). Controlling the false discovery rate: a practical and powerful approach to multiple testing. J. Roy. Stat. Soc.: Ser. B (Methodol.).

