# Supplementary material for: Leukocyte glucose index as a novel biomarker for COVID-19 severity
Source: Sci Rep. 2022 Sep 2;12:14956. doi: 10.1038/s41598-022-18786-5 (PMC9438363; doi:10.1038/s41598-022-18786-5)
Supplement: Supplementary file 1 — Supplementary Information. [file 41598_2022_18786_MOESM1_ESM.docx]

**Leukocyte glucose index as a novel biomarker for COVID-19 severity**

Wendy Marilú Ramos-Hernández^1*,†^, Luis Fernando Soto^2,†^, Marcos Del Rosario-Trinidad^1^, Carlos Noe Farfan-Morales^3^, Luis Adrián De Jesús-González^3^, Gustavo Martínez-Mier^1^, Juan Fidel Osuna-Ramos^4^, Fernando Bastida-González^5^, Víctor Bernal-Dolores^1^, Rosa María del Ángel^3^ and José Manuel Reyes-Ruiz^1,6*^

^1^Unidad Médica de Alta Especialidad, Hospital de Especialidades No. 14, Centro Médico Nacional “Adolfo Ruiz Cortines”, Instituto Mexicano del Seguro Social (IMSS), Veracruz 91897, México

^2^Escuela Profesional de Genética y Biotecnología, Facultad de Ciencias Biológicas, Universidad Nacional Mayor de San Marcos, Lima 15081, Perú

^3^Department of Infectomics and Molecular Pathogenesis, Center for Research and Advanced Studies (CINVESTAV-IPN), Mexico City 07360, Mexico

^4^Escuela de Medicina, Universidad Autónoma de Durango Campus Culiacán, Culiacán Rosales 80050, México

^5^Laboratorio de Biología Molecular, Laboratorio Estatal de Salud Pública del Estado de México, State of Mexico 50130, Mexico

^6^Facultad de Medicina, Región Veracruz, Universidad Veracruzana, Veracruz 91700, Mexico

***Address correspondence to:**

José Manuel Reyes (jose.reyesr@imss.gob.mx)

Wendy Marilú Ramos (dra_ramos_hernandez@hotmail.com)

^†^These authors contributed equally to this work.

**Supplemental Material**

**Supplementary Table S1. Clinical characteristics and laboratory findings in patients with non-severe and severe COVID-19 (Continued).**

| **Variable** | **Severe group (n = 36)** | **Non-severe group (n = 73)** | ***P* value** |
| --- | --- | --- | --- |
| Hypertension, n (%) | 23 (63.89%) | 40 (54.79%) | 0.485 |
| Temperature, °C | 37.37 (0.79) | 36.66 (1.93) | **0.003** |
| HR, beats per minute | 99.47 (20.63) | 92.19 (18.58) | **0.037** |
| PaCO_2_, mmHg | 46.56 (18.41) | 38.97 (13.36) | 0.147 |
| Fever, n (%) | 31 (86.11%) | 55 (75.34%) | 0.295 |
| Cough, n (%) | 34 (94.44%) | 67 (91.78%) | 0.911 |
| Headache, n (%) | 26 (72.22%) | 47 (64.38%) | 0.547 |
| Dyspnea, n (%) | 36 (100%) | 68 (93.15%) | 0.262 |
| Myalgia, n (%) | 12 (33.33%) | 8 (10.96%) | **0.010** |
| Odynophagia, n (%) | 21 (58.33%) | 27 (36.99%) | 0.056 |
| Basophiles, x10^9^/L | 0.6 (2.81) | 0.43 (1.97) | 0.192 |
| Platelets, x10^9^/L | 232.14 (92.68) | 261.45 (113.7) | 0.168 |
| Hematocrit, % | 32.19 (7.87) | 36.95 (8.68) | **0.001** |
| MCV, fL | 90.58 (5) | 90.55 (5.52) | 0.795 |
| BUN, mg/dL | 31.11 (21.91) | 28.85 (22.54) | 0.300 |
| Urea, mg/dL | 61.64 (42.08) | 106.05 (421.41) | 0.214 |
| Cr, mg/dL | 2.98 (3.67) | 2.19 (3.94) | 0.120 |
| Sodium, mmol/L | 138.25 (5.17) | 135.73 (4.49) | **0.006** |
| Potassium, mmol/L | 4.45 (1.11) | 4.25 (0.75) | 0.342 |
| Chloride, mmol/L | 103.92 (7.12) | 102.88 (6.2) | 0.963 |
| BUN/Cr ratio | 21.26 (19.55) | 21.22 (14.87) | 0.759 |

Continuous variables are represented as mean with standard deviation (SD) and discrete variables are represented as number or proportion (%). Statistically significant *P* values (<0.05) are highlighted in bold. Parameters that remained significant after considering gender are indicated with asterisk in the *P* value (*).

HR: heart rate; PaCO_2_: partial pressure of carbon dioxide; MCV: mean corpuscular volume; BUN: blood urea nitrogen; Cr: creatinine.

**Supplementary Table S2. Risk factors associated with severity in female and male patients.**

| **Female** | | | | |
| --- | --- | --- | --- | --- |
| **Predictor** | **OR [95% CI]** | ***P* value** | ***P* *_adjusted_*** | **AUC [95% CI]** |
| ROX index | 0.704 [0.553 - 0.827] | 0.0004 | **0.006** | 90.91 [82.977 – 98.840] |
| SaO_2_, % | 0.869 [0.783 - 0.932] | 0.001 | **0.019** | 88.23 [79.295 – 97.165] |
| RR, beats per minute | 1.297 [1.143 - 1.561] | 0,0007 | **0.011** | 87.18 [76.628 – 97.722] |
| SAFI, mmHg | 0.985 [0.975 - 0.992] | 0.0007 | **0.010** | 86,44 [76.675 – 96.214] |
| PAFI, mmHg | 0.987 [0.977 - 0.994] | 0.002 | **0.031** | 81,17 [69.415 – 92.921] |
| LGI | 2.694 [1.575 - 5.283] | 0.001 | **0.017** | 78,25 [64.284 – 92.208] |
| HCO_3_^-^, mmol/L | 0.838 [0.735 - 0.926] | 0.002 | **0.030** | 76,54 [63.462 – 89.622] |
| **Male** | | | | |
| **Predictor** | **OR [95% CI]** | ***P* value** | ***P* *_adjusted_*** | **AUC [95% CI]** |
| Leukocytes, x109/L | 1.359 [1.145 - 1.696] | 0.001 | **0.025** | 82.38 [69.991 – 94.770] |
| pH | 0.000002 [0.00000000005 - 0.0005] | < 0.001 | **0.010** | 82.14 [68.359 – 95.926] |
| ROX index | 0.816 [0.700 - 0.919] | 0.002 | **0.043** | 80.71 [67.518 – 93.9101] |
| Hemoglobin, g/dL | 0.610 [0.439 - 0.796] | < 0.001 | **0.013** | 80.32 [65.434 – 95.199] |
| SpO_2_, % | 0.925 [0.875 - 0.964] | 0.001 | **0.020** | 78.33 [61.425 – 95.240] |
| RR, beats per minute | 1.181 [1.073 - 1.332] | 0.002 | **0.033** | 74.52 [55.866 – 93.181] |

Statistically significant *P* values (<0.05) are highlighted in bold. ROX index: respiratory rate oxygenation index; SaO_2_: oxygen saturation; RR: respiratory rate; PAFI: PaO_2_/FiO_2_; SAFI: SaO_2_/FiO_2_; LGI: leukocyte glucose index; HCO_3_^−^: arterial bicarbonate; pH: potential hydrogen; SpO_2_: peripherical oxygen saturation.

**Supplementary Table S3. Cut off values of risk factors associated with disease severity in patients with diabetes.**

| **Predictor** | **AUC [95% CI]** | **Cut-off** | **Sensitivity** | **Specificity** | ***P* value** |
| --- | --- | --- | --- | --- | --- |
| ROX index | 0.925 [0.849 – 1] | 9.090 | 0.944 | 0.827 | **< 0.0001** |
| SAFI, mmHg | 0.924 [0.851 - 0.997] | 225 | 0.888 | 0.827 | **< 0.0001** |
| RR | 0.871 [0.769 - 0.974] | 30 | 0.666 | 0.931 | **< 0.0001** |
| SpO_2_, % | 0.840 [0.699 - 0.982] | 86 | 0.777 | 0.896 | **< 0.0001** |
| PAFI, mmHg | 0.851 [0.737 - 0.966] | 167.5 | 0.833 | 0.862 | **0.0001** |
| Leukocytes, x109/L | 0.858 [0.752 - 0.965] | 12 | 0.888 | 0.724 | **< 0.0001** |
| HCO_3_^-^, mmol/L | 0.882 [0.787 - 0.978] | 20 | 0.777 | 0.827 | **< 0.0001** |
| pH | 0.792 [0.649 - 0.935] | 7.33 | 0.722 | 0,827 | **< 0.0001** |
| SaO_2_, % | 0.869 [0.771 - 0.967] | 90 | 1 | 0.586 | **< 0.0001** |
| LGI | 0.915 [0.830 – 1] | 3.15 | 0.833 | 0.931 | **< 0.0001** |
| Hemoglobin, g/dL | 0.791 [0.646 - 0.935] | 11 | 0,722 | 0.896 | **< 0.001** |
| FiO_2_, % | 0.816 [0.697 - 0.935] | 40 | 0.888 | 0.724 | **< 0.001** |

Statistically significant *P* values (<0.05) are highlighted in bold. ROX index: respiratory rate oxygenation index; SAFI: SaO_2_/FiO_2_; RR: respiratory rate; SpO_2_: peripherical oxygen saturation; PAFI: PaO_2_/FiO_2_; HCO_3_^−^: arterial bicarbonate; pH: potential hydrogen; SaO_2_: oxygen saturation; LGI: leukocyte glucose index; FiO_2_: fraction of inspired oxygen.
